# Pathway to Regulatory Approval of Digital Health Technologies in Progressive Supranuclear Palsy: A Scoping Review

**DOI:** 10.3390/brainsci15060587

**Published:** 2025-05-29

**Authors:** Catherine Isroff, Kyurim Kang, Alberto J. Espay, Marian L. Dale, Alexander Pantelyat, Anne-Marie Wills, Chrystalina A. Antoniades

**Affiliations:** 1 Department of Neurology, Mass General Brigham, Boston, MA 02114, USA; 2 Department of Neurology, Johns Hopkins University School of Medicine, Baltimore, MD 21205, USA; 3 James J. and Joan A. Gardner Family Center for Parkinson’s Disease and Movement Disorders Neurology and Rehabilitation Medicine, Department of Neurology, University of Cincinnati, Cincinnati, OH 45219, USA; 4 Department of Neurology, Oregon Health and Science University, Portland, OR 97239, USA; 5 NeuroMetrology Lab, Nuffield Department of Clinical Neurosciences, John Radcliffe Hospital, University of Oxford, Oxford OX1 2JD, UK

**Keywords:** progressive supranuclear palsy, PSP, atypical Parkinsonism, digital health technology, wearable sensor, digital biomarker

## Abstract

**Background/Objectives:** Progressive supranuclear palsy (PSP) is an atypical Parkinsonian disorder characterized by Parkinsonism with gait imbalance, vertical gaze palsy, and frontal cognitive dysfunction. Though digital health technologies (DHTs) are widely used both clinically and in research as outcome measures, there is a lack of consistency applied to these devices and their resulting metrics. This scoping review aims to identify efforts taken to validate wearable DHTs for use in PSP, identify gaps in research, and discuss the steps needed to expand their use and acceptance as primary trial endpoints. **Methods**: In this scoping review, we conducted a search of the MEDLINE database to examine the use of DHTs as outcome measures in Progressive Supranuclear Palsy. **Results**: A total of 17 publications were identified and reviewed. Included articles evaluated the use of DHT to measure lower extremity function/gait, balance, upper extremity function, and speech. **Conclusions**: Our scoping review highlights the importance of standardization of DHT metrics by thorough assessment of their content validity, reliability, construct validity, responsiveness, and discriminant validity. Efforts must be taken to ensure DHTs capture clinically relevant, patient-centered outcome measures that are comparable to conventional rating scales, that consistently discriminate disease progression. Incorporation of DHTs as clinical trial endpoints has the potential to encourage clinical research and to advance patient care.

## 1. Introduction

Progressive supranuclear palsy (PSP) is an atypical Parkinsonian disorder (APD) clinically characterized by Parkinsonism, specifically akinesia, with early and severe gait imbalance, vertical gaze palsy and frontal cognitive dysfunction [1]. Pathologically, PSP is a 4R-tauopathy characterized by hyperphosphorylated tau proteins that deposit in neurons and glial cells leading to neuronal degeneration [2,3]. In PSP, neurofibrillary tangle deposition in neurons is often observed in the globus pallidus, subthalamic nucleus and substantia nigra, while tufted astrocyte accumulation in glial cells is predominantly detected in peri-Rolandic cortices and the putamen [3].

PSP is a progressive and, ultimately, fatal disorder with no effective treatment [1]. The current gold-standard for measuring PSP progression is the Progressive Supranuclear Palsy Rating Scale (PSPRS) [4]. Most clinical trials for PSP currently require in-person assessments with a rater trained in the administration of the PSPRS to quantify outcome measures [5,6].

The U.S. Food and Drug Administration (FDA) defines digital health technology (DHT) as “a system that uses computing platforms, connectivity, software, and/or sensors, for health care and related uses” [7]. There are a wide variety of wearable DHTs available in today’s global market for both clinical and research use [8]. DHTs are often used only as secondary or exploratory endpoints in clinical trials. Though DHTs currently have some limitations, they offer several unique opportunities relative to conventional rating scales (Table 1).

### 1.1. Assessing Validity of Digital Health Technologies

To achieve adoption as a clinical trial endpoint and to meet regulatory requirements, there are several metrics that a DHT must meet, including content validity, reliability, construct validity, responsiveness, and discriminant validity. Content validity, also called technical validity, compares a measurement derived from a DHT with a measurement obtained via conventional methods [13]. Reliability of the sensor implies minimal variance when the same device is used repeatedly to measure a single patient outcome; however, it should also denote minimal inter-device variance [9,13]. Construct validity, also known as clinical validity, suggests that outcome measures have shown comparable results when evaluated against a defined clinical scale [9,13,14]. Responsiveness suggests the endpoint can identify longitudinal changes within a group [13]. In their review of wearable devices for Parkinson’s disease (PD), Del Din et al. also emphasize that a DHT should demonstrate discriminant validity: the ability to differentiate between groups (such as between healthy and control populations) [14].

Finally, the digital biomarker must reflect patient-centered, clinically relevant outcome measures [15]. Mitsi et al. postulate that this question of participant engagement and, ultimately, adherence is the last and possibly most essential form of validation [16].

### 1.2. Process to Obtain Regulatory Approval for DHTs in Other Neurologic Diseases

We examined the regulatory pathway for DHTs in other neurological diseases in order to contextualize this review. The first DHT to obtain EMA regulatory approval for use as a secondary clinical trial endpoint is called ActiMyo^®^, a two-strap-based wearable sensor [17,18]. ActiMyo^®^ was approved for use in Duchenne muscular dystrophy (DMD). To obtain regulatory approval, several steps were taken to validate the technology. Technical validity, also called content validity, was established by comparing DHT and clinical measures of the 6 min walk test, showing only a 5% difference between the two outcomes [17]. Reliability was shown by measuring variance in stride length and velocity demonstrating only 2.2–4.4% variability [17]. Clinical validity, also called construct validity, was also shown through comparison to the North Star Ambulatory Assessment, and the 4-stair climbing test, demonstrating high correlation (0.78 and 0.64, respectively) [17]. In addition, ActiMyo sensors demonstrated responsiveness by tracking individuals’ clinical decline with longitudinal data collection over a 6-month period [17]. This success can inform efforts to streamline regulatory approval of DHTs as clinical trial endpoints in other diseases.

Wearable DHTs have been extensively tested in individuals with PD. The devices currently on the market for use in PD have been ‘FDA-cleared’, which indicates that they demonstrated ‘substantial equivalence’ to another device already on the market. Wearable DHTs are typically categorized by the FDA as class II, or moderate risk devices. As such, the FDA clearance process requires submission of a Premarket Notification via the 510(k) clearance process [19]. In 2021, Del Din et al. summarized DHT motor-specific outcomes for individuals with PD with a goal to establish technical and clinical validation (in this case designated as ‘criterion validity’ and ‘construct validity’) for these devices [14]. By their analysis, several DHTs achieved these validation metrics for use in PD to measure motor symptoms, including tremor and bradykinesia, postural instability and gait disturbance [14]. Despite this, regulatory bodies have approved very few DHTs for use in PD and no DHT has been approved as a clinical trial endpoint in PD.

### 1.3. Objective of This Scoping Review

Based on the gathered information, we decided to conduct further research to analyze the use of DHTs in PSP. The aim of this scoping review is to outline efforts taken to validate wearable DHTs for use in PSP, identify gaps in research, and to discuss the steps needed to expand their use and acceptance as primary trial endpoints by United States and European regulatory agencies.

## 2. Materials and Methods

This review follows the guidelines outlined in the Preferred Reporting Items for Systematic Review and Meta-Analysis (PRISMA) Extension for Scoping Reviews [20].

The review was conducted using the MEDLINE database. The review began in February of 2025 and was completed in March of 2025 with most recent search performed on 23 March 2025. The search was conducted using the keywords outlined in Table 2. An additional search filter for a 15-year date restriction was applied for inclusion in the study. All searches were performed in English.

**Figure 1:**
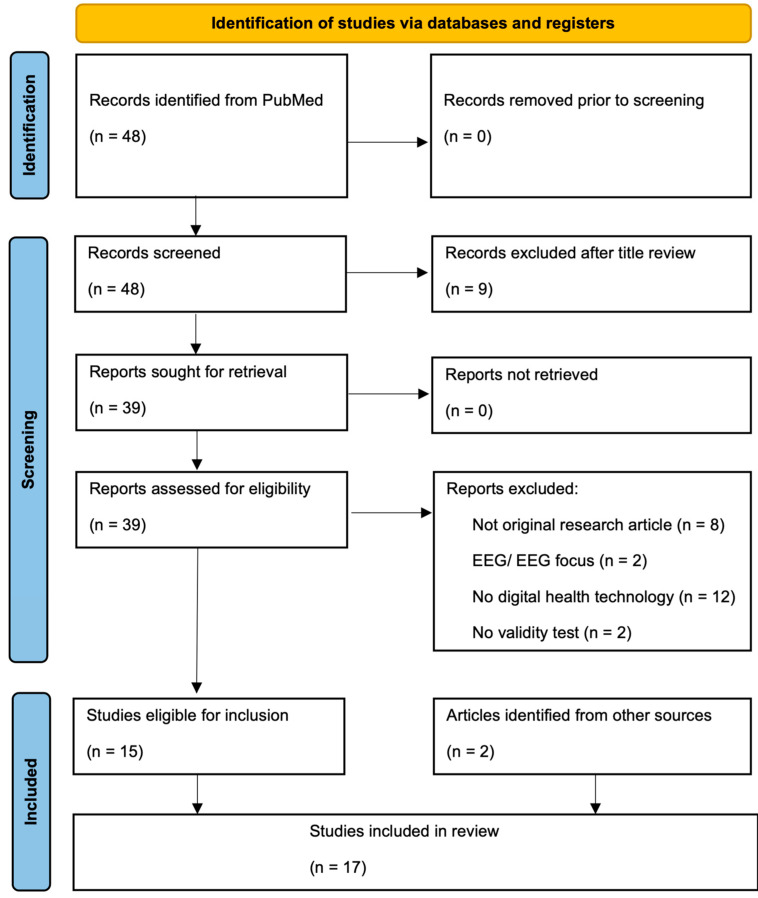
PRISMA flow diagram.

This review was conducted by first assessing the titles of the papers, followed by the abstracts, and finally the full papers. Only English-language articles were included. The review included studies with participants from all age groups within the PSP population. Only original research articles, including peer-reviewed journal articles and conference papers, were included in the review. Articles on digital health technology as an outcome measure in individuals with PSP were included. Studies that focused solely on electroencephalogram or electrocardiogram data were also excluded. Studies with and without comparison groups (e.g., healthy controls, other movement disorders) were included in this review. Studies utilizing DHT-derived endpoints as outcome measures were included in the review. Studies that did not address validity, reliability, or responsiveness were excluded. Finally, an additional step to evaluate the reference list of included articles was performed to locate other articles that were not included in the database search but could potentially be eligible for inclusion in the review. The selection process is displayed in a PRISMA flowchart (see Figure 1). Data charting was performed independently by C.I.

A complete synthesis of the results is conducted in tabular and descriptive form in the Results section.

## 3. Results

The entire article selection process can be reviewed in the PRISMA flowchart (Figure 1). The search criteria initially identified 48 articles. A total of 9 articles were excluded after reviewing the titles for eligibility, resulting in 39 articles. All reports were retrieved. A total of 15 articles were eligible for inclusion after full-text assessment. Two additional eligible articles were identified from other resources including assessment of included reference lists and dedicated search for articles evaluating the previously described sensors. In summary, a total of 17 articles were included in this scoping review.

The included studies were published between 2014 and 2024 with 12 of the 17 studies published since 2019, underlining growing interest in this area. All studies were published in peer-reviewed journals. Three of the articles included more than one validation technique [21,22,23]. The sensors evaluated include the Opal IMU Sensor (APDM, Portland, OR, USA), SHIMMER 2 Sensor (Shimmer, Dublin, Ireland), LEGSys+ Sensors (BioSensics LLC, Newton, MA, USA), Balance Master Clinical Research System (NeuroCom International, Clarckamas, OR, USA), activPAL accelerometer (PAL Technologies Ltd., Glasgow, UK), IMSUs from the University of Belgrade, Serbia (STMicroelectronics, Geneva, Switzerland), BioDigit Speech Sensor (BioSensics LLC, Newton, MA, USA), ki: SB-M intelligibility score (ki:elements, Saarbrücken, Germany), and the Speech Activity Detector (University of Pennsylvania Linguistic Data Consortium, Philadelphia, PA, USA). Details and outcomes from the qualified scientific papers can be found in Table 3.

Nine of the seventeen studies [21,22,23,24,25,26,27,28,29] in this review utilized the 2017 MDS diagnostic criteria [30] for inclusion of PSP participants (either probable or possible PSP), while six [31,32,33,34,35,36] used the older 1996 NINDS-SPSP criteria [37] for inclusion in the studies. Two of the included studies did not specify the PSP diagnostic criteria used [38,39]. Most studies did not specify PSP predominance type, but three studies reported inclusion of only PSP-Richardson’s syndrome or PSP-Parkinsonism predominant variants [24,25,28].

This review determined that there are a few notable gaps in the assessment of the use of wearable DHTs in progressive supranuclear palsy. Some sensors have demonstrated content validity in PSP; however, others have only been technically assessed in healthy control populations or in Parkinson’s disease [40,41]. Currently the FDA recommends that the DHT be assessed in the proposed participant population [7]. Additionally, the review highlights the paucity of non-motor DHT-derived outcomes with only three studies highlighting speech assessment through the use of DHT [22,23,29]. The search revealed no articles assessing oculography even though ocular motor dysfunction is a core clinical feature of PSP as defined by the Movement Disorder Society 2017 Criteria [30].

While excluded from this review due to lack of validity testing, a paper by Ohara et al. is novel as it utilizes DHT data as the primary outcome measure [42]. The study assessed response to spinal tap vs. sham spinal tap procedures in PSP, normal pressure hydrocephalus (NPH), and healthy controls [42]. DHT was used in assessment of timed up-and-go test and showed that the ratio of tap responders and sham tap responders in PSP was not statistically different from those with NPH but was different from the response seen in HC [42].

Many of the included studies assess the construct validity and responsiveness of the sensors relative to the PSPRS to demonstrate the non-inferiority of the DHT-derived data. Importantly, Sotirakis et al. report that their linear regression model, which incorporates three sensor-measured gait parameters, detected a quantifiable change from baseline three months earlier than the PSPRS [25]. This suggests the potential for DHTs to yield outcome measures that are more discriminant than conventional rating scales.

**Table 3:** Measurement properties and study outcomes of several DHTs in PSP.

Study/ Year	Sensor	Country	Type of Participants (n)	Measurement Property Assessed	Outcomes
Lower Extremity Function/Gait					
Klenk et al., 2016 [31]	activPAL3 accelerometers (PAL Technologies Ltd., Glasgow, UK)	Germany	DA (34), PSP (15), PD (16), HC (38)	Discriminant validity	Significant decrease in average daily number of walking bouts and number of sit-to-stand transfers per day in PSP group compared to HC [31].
Raccagni et al., 2018 [33]	SHIMMER 2 Sensors (Shimmer, Dublin, Ireland)	Austria, Germany	PD (25), MSA (13), PSP (12)	Construct validity	Significant correlation between total PSPRS score and sensor-measured stride length (SCC 0.682, *p*-value 0.021) [33].
Gassner et al., 2019 [32]	SHIMMER 2 Sensors (Shimmer, Dublin, Ireland)	Austria, Germany	PD (40), APD 20 (MSA-p (11), PSP (9)	Discriminant validity	Statistically significant difference between PD and APD groups in sensor-based calculation of stride length, gait velocity, and toe-off angle [32].
De Vos et al., 2020 [24]	Opal IMU Sensors (APDM, Portland, OR, USA)	UK	PSP (21) PD (20), HC (39)	Discriminant validity	Comparison of data from two-minute walk, static sway test, and timed up-and-go task using the Random Forest machine learning algorithm resulted in discrimination of PSP from PD with 86% sensitivity and 90% specificity, and PSP from HC with 90% sensitivity and 97% specificity [24].
Sotirakis et al., 2022 [25]	Opal IMU Sensors (APDM, Portland, OR, USA)	UK	PSP (17)	Responsiveness	Significant longitudinal differences in a linear regression model incorporating sensor-measured mean turn velocity, standard deviation of stride length, and mean toe-off angle with ability to detect statistically significant progression 3 months earlier than clinical scores [25].
Ricciardi et al., 2023 [26]	Opal IMU Sensors (APDM, Portland, OR, USA)	Italy	PSP (15)	Content validity	Sensor compared against optoelectronic measurement of gait showing concordance in gait speed (slope of Passing–Bablok regression line of 1.02 and intercept of 0.05), but systematic error in measurement of cadence and cycle duration [26].
Abate et al., 2023 [21]	Opal IMU Sensors (APDM, Portland, OR, USA)	Italy	PSP (35)	Construct validity	Inverse correlation between PSPRS total score and sensor-measured gait speed (r = −0.434; *p* < 0.001) and turning velocity (r = −0.579; *p* < 0.001) in 2 min walk test. Positive correlation between PSPRS total score and sensor-measured turn duration (r = 0.411; *p* < 0.001) in 2 min walk test [21].
				Responsiveness	Significant change in sensor measured gait cadence and cycle duration during 2 min walk test over 3 month follow-up [21].
Sharma et al., 2023 [27]	LEGSys+ Sensors BioSensics LLC, Newton, MA, USA)	USA	PSP (11), PD (12)	Construct validity	Correlation between virtually administered PSPRS score and sensor-measured Sit-to-Stand Transition time in Timed Up and Go test (SCC 0.84, uncorrected *p*-value 0.005) [27].
Balance/Falls					
Baston et al., 2014 [38]	Opal IMU Sensors (APDM, Portland, OR, USA)	USA	PD (5), PSP (7)	Discriminant validity	PD and PSP subjects showed a predominant ankle strategy, unlike the HC group, but PSP subjects were not able to reduce sway area resulting in several falls for PSP group [38].
Dale et al., 2017 [34]	Balance Master Clinical Research System (NeuroCom International, Clarckamas, OR, USA), Opal IMU Sensors (APDM, Portland, OR, USA)	USA	PSP (12), PD (12), HC (12)	Discriminant validity	Individuals with PSP were less able than PD or HC counterparts to perceive toes-up platform tilts and exhibited fewer corrective motor responses in reaction to forward platform translations and toes-up surface tilts [34].
Srulijes et al., 2019 [35]	activPAL accelerometers (PAL Technologies Ltd., Glasgow, UK)	Germany	DA (31), PSP (12), PD (14), HC (31)	Discriminant validity	Physical activity measured via accelerometer was compared to recorded fall incidence. The PSP group with high walking bout length showed a significantly higher fall incidence of 45.3 falls/person years compared to the low-activity group 12.5 falls/person years [35].
Upper Extremity Function					
Djurić-Jovičić et al., 2016 [36]	IMSUs from the University of Belgrade, Serbia (STMicroelectronics, Geneva, Switzerland)	Serbia	PD (13), PSP (15), MSA (14), HC (14)	Construct validity	Significant correlation between sensor-measured amplitude (ρ = −0.73; *p* = 0.007) and speed slope (ρ = −0.69; *p* = 0.012) of finger tapping as compared to the FAB total score [36].
Bobić et al., 2019 [39]	IMSUs from the University of Belgrade, Serbia (STMicroelectronics, Geneva, Switzerland)	Serbia	PD (13), MSA (17), PSP (14), HC (12)	Content validity	Sensor-generated measure of bradykinesia (overall score computed by algorithm) was directly compared to movement disorder physicians’ rating of bradykinesia with an overall accuracy of 83.76 ± 7.86% in individuals with PSP [39].
Belić et al., 2023 [28]	IMSUs from the University of Belgrade, Serbia (STMicroelectronics, Geneva, Switzerland)	Serbia	PD (14), PSP (16), MSA (13), HC (11)	Discriminant validity	Using sensors to evaluate finger tapping data resulted in correct identification of 11 of the 16 individuals with PSP (2 individuals with PSP incorrectly identified as MSA, and 3 as HC) [28].
Speech					
Parjane et al., 2021 [23]	Speech Activity Detector (University of Pennsylvania Linguistic Data Consortium, Philadelphia, PA, USA)	USA	PSPS-CBS (87), naPPA (25), HC (41)	Construct validity	Longer pause segment duration and lower speech rate correlated with phonemic fluency score in PSPS-CBS; however, no correlation between sensor outcomes and other standard neuropsychological assessment [23].
				Discriminant validity	PSPS-CBS had statistically significant shorter speech segments, longer pause segments, higher pause rate, and reduced f0 range compared to HC [23].
Kang et al., 2023 [29]	BioDigit Speech Sensor (BioSensics LLC, Newton, MA, USA)	USA	PSP (11), PD (10)	Construct validity	Negative correlation between PSPRS dysphagia score and sensor-derived similarity dynamic time warping in rainbow passage reading (r 0.78, *p* 0.007). Positive correlation between PSPRS dysphagia score and sensor-derived ratio of extra words (r 0.82, *p* 0.004) and ratio of missing words (r 0.78, *p* 0.007) in rainbow passage reading. Positive correlation between PSPRS dysarthria and bulbar scores and sensor-derived articulation rate in reverse number count assessment [29].
Tröger et al., 2024 [22]	ki: SB-M intelligibility score (ki:elements, Saarbrücken, Germany)	Czech Republic, Columbia, Germany	Czech: HD (39), PD (43), ALS (16), PSP (17), HC (46); Colombian: HC (50), PD (50); German PD (98)	Reliability	Comparison of two different automatic speech recognition systems as basis for SB-M intelligibility score resulted in ICC of 0.841 [22].
				Construct validity	Non-statistically significant correlation (suspected to be due to small sample size) in ki: SB-M intelligibility score and NNIPPS (r = −0.42, *p* < 0.10, d = 0.92) [22].

Healthy controls (HC), Multiple system atrophy (MSA), Spearman’s correlation coefficient (SCC), Frontal Assessment Battery (FAB), Progressive Supranuclear Palsy (PSP), Progressive Supranuclear Palsy Rating Scale (PSPRS), Parkinson’s disease (PD), Degenerative ataxia (DA), Amyotrophic Lateral Sclerosis (ALS), Huntington’s disease (HD), Progressive supranuclear palsy syndrome (PSPS), corticobasal syndrome (CBS), non-fluent/agrammatic primary progressive aphasia (naPPA), Inertial Measurement Unit (IMU), Inertial Measurement Sensor Units (IMSUs), Intraclass correlation coefficient (ICC), Speech biomarker score for motor speech disorders (SB-M), Natural History and Neuroprotection in Parkinson Plus Syndromes-Parkinson Plus Scale (NNIPPS).

## 4. Discussion

This paper maps the efforts to substantiate various DHTs for use in PSP through a scoping review of 17 studies. As shown above, several DHTs have demonstrated content validity, reliability, construct validity, responsiveness, and discriminant validity in PSP to capture gait, balance, upper extremity function, and speech data. These outcome measures can be used alone or in combination to generate insight into PSP disease severity.

There are several limitations to our review. First, PSP is a heterogeneous disease [43]. Only two major subtypes—PSP-Richardson’s syndrome and PSP-Parkinsonism predominant—were included in most of these studies. Additionally, the sample size of all included DHT studies was low, raising concerns for generalizability of the data.

In addition, our search criteria did not capture studies focused on oculography. As ocular motor dysfunction is a core feature of PSP, oculography could lend additional insight into disease severity and further validate DHT data for use in clinical trials.

Currently, DHTs attempt to replicate measures derived via conventional methods. As the technology continues to advance, regulators will need to decide (1) whether this comparison remains valid and (2) how to treat digital biomarkers with no conventional comparator.

In order for DHTs to be accepted as clinical trial endpoints in PSP, they must first demonstrate responsiveness and the ability to capture disease progression and longitudinal change. Abate et al. demonstrated a significant longitudinal change in DHT outcome measures of gait at 3 months, suggesting that these wearable sensors can quantify progression of gait changes in PSP [21]. The use of DHTs as a primary outcome measure in the study by Ohara et al. suggests the feasibility of including DHT-derived data as a primary endpoint [42]. However, more studies are needed to corroborate DHT’s sensitivity to progressive change at additional timepoints.

Interestingly, Sotirakis et al. demonstrate that the longitudinal difference in gait captured by DHT is detectable up to three months earlier than by the PSPRS alone [25]. Earlier detection of clinical differences in clinical trials could lead to a positive trial outcome in a disease that otherwise has limited treatments. However, studies have not yet shown that these early changes identified by DHT are clinically meaningful to patients, caregivers and clinicians [7]. Demonstrating clinical meaningfulness is a critical step, not only to obtain regulatory approval of these devices, but also to ensure patient adoption of the studied intervention.

Despite these caveats, there are many potential advantages to adopting DHT’s as clinical outcome measures. DHTs have the potential to provide more frequent, standardized, and quantitative assessments. Ultimately, this precision implies smaller, more efficient, and cost-effective clinical trials in the future. DHTs also offer the ability to assess participants in a remote, home-based, real-world setting [9,44]. The remote nature of DHT assessments also offer wider inclusivity for patients who are geographically or financially limited. For these reasons, it is of the utmost importance that regulatory bodies continue to assess the use of these technologies as clinical trial endpoints in PSP. Assessment of content validity, reliability, construct validity, responsiveness, and discriminant validity for these DHTs in PSP should provide additional evidence to the regulatory agencies towards acceptance as clinical trials endpoints.

## Figures and Tables

**Table 1:** Current limitations and opportunities of digital health technologies.

Limitations	Opportunities
DHTs often capture a single disease metric, not necessarily a global picture of an individual’s disease [9].	DHTs provide more continuous, objective and reliable measures in comparison with ordinal rating scales, as has been shown in PD [10,11].
DHTs characterize motor dysfunction but often neglect non-motor aspects of an individual’s disease [9].	DHTs allow for remote data collection and expand access to individuals who may be geographically or economically separated from academic medical centers [12].
DHTs produce excessive data requiring the user to sort through acquired data [9].	DHTs may provide more ecologically valid assessments by measuring function in a low-stress, home environment as compared to hospital-based evaluations [12].
Use and adoption of DHTs may be limited by technological literacy.	Improved healthcare accessibility via DHT use leads to inclusivity and enrollment of cohorts more representative of the general population [12].

**Table 2:** MEDLINE search terms.

Term 1		Term 2
progressive supranuclear palsy	AND	digital health technology
		digital technology
		digital health
		DHT
		sensor

## Data Availability

No new data were created or analyzed in this study. Data sharing is not applicable to this article.

## Abbreviations

The following abbreviations are used in this manuscript:

DHT	Digital health technologies
PSP	Progressive Supranuclear Palsy
PSPRS	Progressive Supranuclear Palsy Rating Scale
PD	Parkinson’s disease
DA	Degenerative ataxia
ALS	Amyotrophic Lateral Sclerosis
HD	Huntington’s disease
MSA	Multiple system atrophy
naPPA	Non-fluent/agrammatic primary progressive aphasia
CBS	Corticobasal syndrome
HC	Healthy controls
DMD	Duchenne muscular dystrophy
EMA	European Medicines Agency
FDA	Food and Drug Administration
FAB	Frontal Assessment Battery (FAB)
IMU	Inertial Measurement Unit
IMSUs	Inertial Measurement Sensor Units
LCC	Lin’s concordance correlation coefficient
PCC	Pearson correlation coefficient
SCC	Spearman’s correlation coefficient
ICC	Intraclass correlation coefficient
SB-M	Speech biomarker score for motor speech disorders (SB-M)
NNIPPS	Natural History and Neuroprotection in Parkinson Plus Syndromes—Parkinson Plus Scale
